# Relating the Bipolar Spectrum to Dysregulation of Behavioural Activation: A Perspective from Dynamical Modelling

**DOI:** 10.1371/journal.pone.0063345

**Published:** 2013-05-14

**Authors:** Arno Steinacher, Kim A. Wright

**Affiliations:** 1 Systems Biology Group, College of Engineering, Mathematics, and Physical Sciences, University of Exeter, Exeter, United Kingdom; 2 School of Psychology, Washington Singer Labs, University of Exeter, Exeter, United Kingdom; University of Chicago, United States of America

## Abstract

Bipolar Disorders affect a substantial minority of the population and result in significant personal, social and economic costs. Understanding of the causes of, and consequently the most effective interventions for, this condition is an area requiring development. Drawing upon theories of Bipolar Disorder that propose the condition to be underpinned by dysregulation of systems governing behavioural activation or approach motivation, we present a mathematical model of the regulation of behavioural activation. The model is informed by non-linear, dynamical principles and as such proposes that the transition from “non-bipolar” to “bipolar” diagnostic status corresponds to a switch from mono- to multistability of behavioural activation level, rather than an increase in oscillation of mood. Consistent with descriptions of the behavioural activation or approach system in the literature, auto-activation and auto-inhibitory feedback is inherent within our model. Comparison between our model and empirical, observational data reveals that by increasing the non-linearity dimension in our model, important features of Bipolar Spectrum disorders are reproduced. Analysis from stochastic simulation of the system reveals the role of noise in behavioural activation regulation and indicates that an increase of nonlinearity promotes noise to jump scales from small fluctuations of activation levels to longer lasting, but less variable episodes. We conclude that further research is required to relate parameters of our model to key behavioural and biological variables observed in Bipolar Disorder.

## Introduction

Mood disorders such as bipolar disorder have not yet attracted substantial interest in the community of dynamical modelling. This is surprising, since bipolar disorder is one type of affective disorder exhibiting strikingly complex switch-like dynamics between normal, depressive and manic or hypomanic states. These state transitions may be regular, but may also lead to chaotic behaviour. Diagnostically, several forms of Bipolar Disorder exist, including: Bipolar I Disorder (BD-I), often considered to be the most severe form of the disorder and the only one to include presence of full manic episodes; Bipolar II Disorder (BD-II), which comprises both hypomanic and major depressive episodes; Cyclothymia, which involves periods of hypomania and minor depression over at least two years with little time spent in a euthymic state; variants of Bipolar Disorder classified as Bipolar Disorder not otherwise specified, which involve fluctuations in levels of depressive and hypomanic symptoms that are not sufficiently severe or prolonged to represent full affective episodes, yet fall outside the person’s normal range of behaviour [1]. Bipolar disorder is a considerable public health problem. Worldwide, the estimated prevalence of ilnesses from the bipolar spectrum is an estimated 2.4% [2]. Compared to the population average, patients suffering from bipolar disorder have a 12.3 times higher rate of suicide [3]. Moreover, bipolar disorder is also associated with increased risks of other illnesses, such as coronary heart disease or cancer [4].

Given that the precise cause of this illness is not yet known, and there is considerable room for improvement in terms of treatment [5], dynamical modelling seems to be a useful way to integrate the knowledge from many levels of research and rigorously test hypotheses of our current understanding of this illness. The pursuit of such an approach has so far been hindered by lack of suitable data, and only a few attempts have been made in this direction. In one mathematical modelling study, bipolar disorder is described in terms of oscillatory behaviour of emotional states using a van der Pol oscillator [6]. In this model, a stable limit cycle is reached at the onset of the illness, which can be reduced in amplitude upon treatment. Aside from this deterministic approach, there are also a few models dealing with stochasticity in affective disorders and the role of noise in episode sensitisation [7]–[9]. These studies implement aspects of the kindling model, which is used in several neuropsychiatric contexts [10], describing the progression from externally induced disease episodes to autonomously occurring episodes following sensitisation. Modelling a positive feedback between sensitisation and an unspecific disease system, these studies provide a conceptual understanding and are able to reproduce some phenomena known from bipolar disorder such as transient events and chaotic behaviour.

One recently published mathematical model describes bipolar disorder by means of a dynamical system of a double negative feedback loop which gives rise to bistability [11]. Similar to previous deterministic approaches, it limits itself to the description of sustained oscillations between extreme mood states. However, while mood is often alternating in bipolar disorder, there is evidence based on the investigation of longitudinal studies that this alternation it is not truly oscillatory but is instead directed by chaotic attractors [12], [13]. Moreover, patients typically exhibit often quite extended episodes of normal mood between manic and depressive episodes. These intermediate phases and the events triggering a transition to depressive or hypomanic/manic episodes are of high interest for clinical treatment.

In this paper, we present a minimal mathematical model of mood regulation in bipolar disorder, implementing hypotheses regarding the auto-regulatory nature of the Behavioural Activation/Approach System (BAS), and predicting that increasing nonlinearity in this system leads to multistability and switch-like transistions between activation or engagement levels in an individual. This model is informed by data on BAS activity in bipolar patients and is able to reproduce some typical dynamics of bipolar disorder, such as a slower recovery time after frustrating or rewarding events in bipolar patients. This effect has been associated with the number of previous episodes in empirical studies [14]. In our model this can be reproduced by increasing the nonlinearity parameter 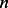.

The BAS promotes active engagement with the environment following signals of reward [15]–[18]. With increasing BAS activity, an individual experiences increased cognitive activity that aims towards achieving goals and approach behaviours, corresponding to positive emotions (such as motivation or elevated mood), but also potentially irritability and anger if goal progress is thwarted [15], [19], [20]. The BAS is mainly activated by rewarding stimuli, such as food, social contact, sex or novelty. Following such stimuli, it increases further engagement with the environment. Typical behaviours associated with high BAS levels are high energy, locomotion and motivation [15]. Neurobiologically, the BAS is thought to be related to the dopaminergic reward pathways [15], [16]. There is evidence from electroencephalographic and neuroimaging data that higher relative activity in the left prefrontal cerebral cortex is associated with approach-related motivation [21], possibly implicating it in the BAS circuitry. There are a number of studies supporting the idea that hypomanic/manic and depressive symptoms in bipolar spectrum disorders are both related to hypersensitivity of the BAS [15], [22]–[25], however the mechanistic basis of such hypersensitivity is still unclear.

Here, we define the BAS to act as an auto-activation system, controlling behavioural activity or engagement and exhibiting inherent properties of nonlinearity. Our minimal model shows that increase in nonlinearity of this system alone is sufficient to model the transition from normal-type activity dynamics to those found in bipolar patients. With the addition of an auto-inhibitory component, this system is multistable at higher degrees of nonlinearity. We can show that the continuous transition between normal-type and bipolar-type dynamics is due only to variation in the nonlinearity parameter in the system.

This hypothesis is directly informed by an empirical study on behavioural dynamics in bipolar patients which shows that time taken for behavioural activation levels to recover from rewarding or frustrating events increases with increasing number of manic and bipolar episodes respecitvely [14]. Data from this study have been re-analysed and the model has been scaled to fit these empirical findings. Deterministic and stochastic simulations of the model have been implemented using numerical integration of the system. Results from the stochastic simulations elucidate the roles of extrinsic and intrinsic noise in an individual’s development of bipolar disorder. As known from other control systems based on positive feedback, nonlinearity tends to increase noise in the system. Our results indicate that increasing noise levels lead to a transition from lower-scale variability (local fluctuations in activation levels around a steady state) to higher-scale variability (occurrence of episodes with lower local fluctuations).

## Methods

### Model Formulation

The model has one variable, 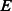, which stands for the engagement or level of behavioural activation and describes the propensity of an individual to interact with its environment. It should be noted at this point that while high activation levels might correlate with positive or elevated mood and low activation levels correlate with low moods, our intention is not to model mood states, but to specifically model behavioural activation/approach levels, which have been shown to correlate with manic, hypomanic and depressive episodes in bipolar disorder [22], [23], [26], [27]. By doing so, we allow for the occurrence of so-called mixed states [28], and moreover account for the finding that manic or hypomanic episodes can manifest themselves as increase in irritability or anger, which can be an output of high BAS activity [20].

**Figure 1:**
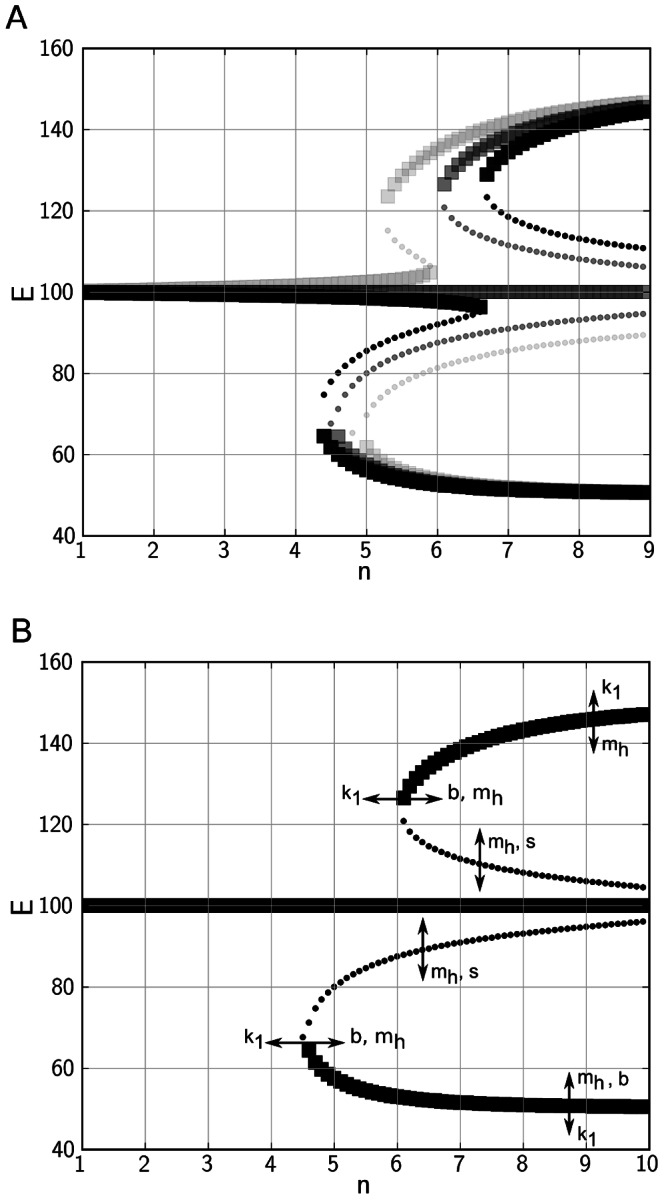
Bifurcation diagram of the system, showing a saddle-node bifurcation; and susceptibility to parameter changes. Thick squared markers signify stable branches of the system, smaller dots signify unstable branches. A: increasing values of 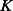 are corresponding to darker colors. B: Change in model parameters and their effects on shifting branches in the bifurcation diagram. Increasing a parameter is indicated by arrow direction.

**Figure 2:**
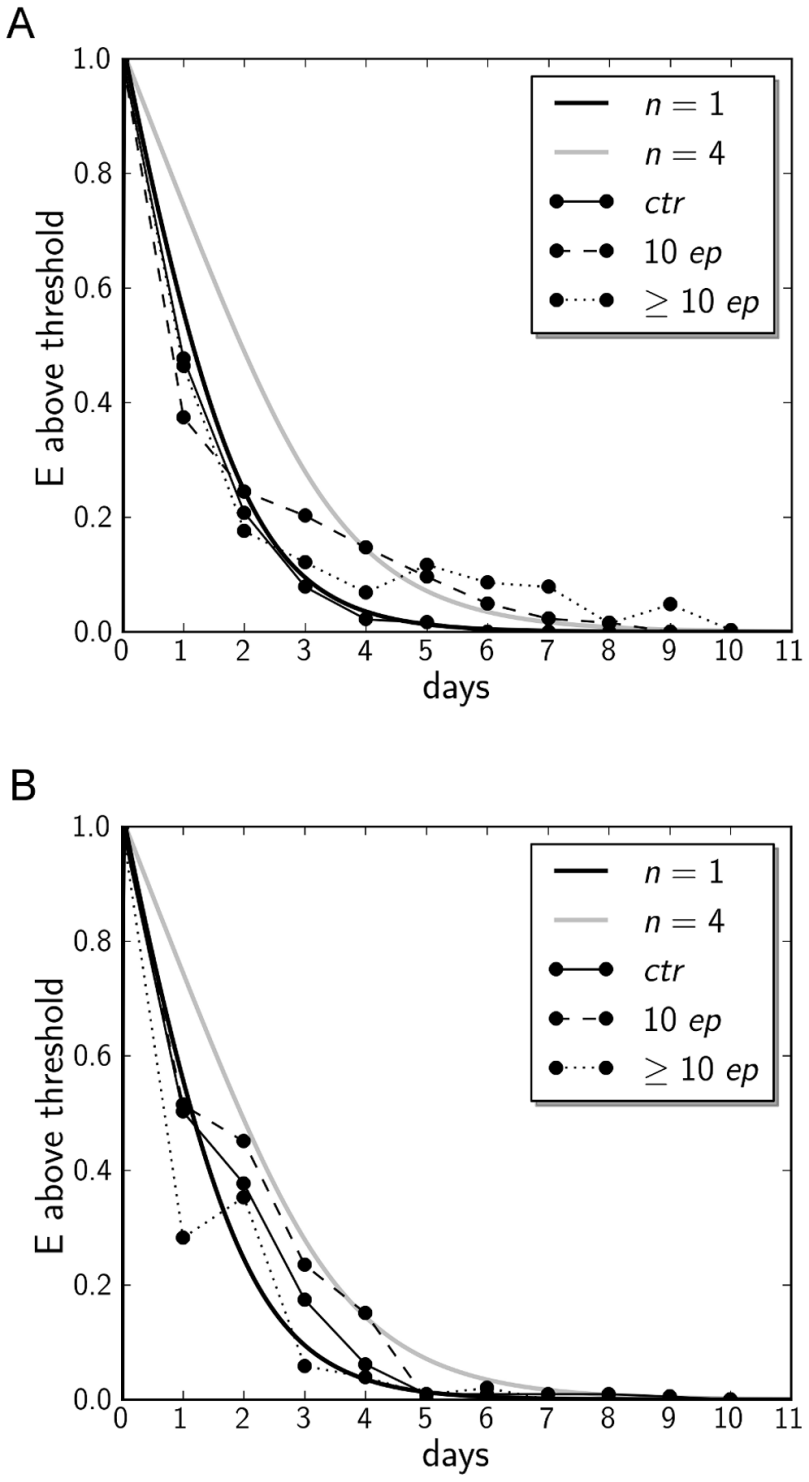
Return of mood to baseline after activating and inhibiting events. A: return to baseline levels after activating events. B: return to baseline levels after inhibiting events. Empirical data (thick black lines) are compared to simulation data (coloured lines). BES (Behavioural Engagement Scale) levels are normalized to maximum value after disturbance of reward and frustration. Zero on the y axis corresponds to the baseline BES level. Solid black line: Control group (N = 18 for reward, N = 51 for frustration plot) Dashed black line: Patients with less than 10 previous episodes. (N = 8 for reward, N = 26 for frustration plot) Dotted black line: Patients with 10 previous episodes or more. (N = 7 for reward, N = 14 for frustration plot). For the simulation data: Black and gray lines correspond to 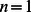 and 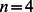, respectively. N represents the sample size.



The regulation of 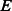 is here understood as being regulated by two feedbacks: one auto-activation feedback, mirroring the BAS and incorporating the function of self-activation, expressed as the up-regulation of 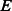 by itself, and an additional auto-regulatory negative feedback, which stands for a tendency to keep 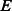 at normal levels. Here, 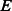 is treated analogously to a substance being produced and degraded in certain processes. This mathematical formalism is very common in theoretical approaches to describe regulatory networks on molecular or cellular levels, such as in gene expression or metabolic networks [29]. Since we are interested only in the dynamical relationship of the variables of interest at a very high organisational level, without attempting to capture the exact biological underpinnings of these phenomena, we borrow the terminology of systems biology to describe BAS regulation. This approach allows us to minimise the level of complexity in the system and focus on specific dynamics of interest, which in this case is the role of nonlinearity in mood regulation. The system is open, such that there is a constant influx of 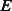 at the rate 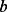, and a decay of 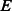 with the rate 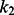. The constant influx rate 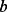 determines the baseline level of the fixed point, especially for the lower fixed point in the region of multistability at high 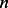. Thus, if 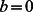, the lower fixed point at high 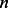 would be 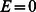, which corresponds to a non-existing level of activation. While this would not change the system behaviour qualitatively, we chose 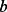 to be a positive rate to ensure a less extreme steady state level for 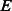. Overall, this can be written as
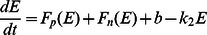
(1)where 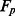 stands for the positive feedback and 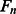 stands for the negative feedback in which 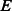 is involved. Auto-activation of 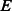 is expressed as a Hill function, such that
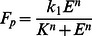
(2)with 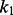 being the maximum rate of 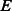 ‘production’, 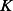 being the rate at which 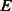 production is half of the maximum rate, and 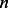 being the nonlinearity parameter. In molecular systems, 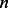 usually corresponds to the cooperativity of an enzyme. In our case, it could be interpreted as the effectiveness of the system to activate 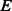, with 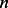 defining the shape of its activation curve 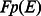. Thus, low levels of nonlinearity lead to hyperbolic response dynamics of 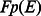, whereas high levels of nonlinearity lead to a sigmoidal-shape ultrasensitive response (see fig. S1) [30]. As a consequence, at certain levels of 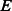 the responsiveness of the activation is low, whereas at a given level determined by the parameter K the responsiveness is high. This renders certain parts of the mood regulation system more sensitive than others. This is in line with the idea that the BAS is poorly regulated in individuals with bipolar disorder, linking depression to an inactive BAS and mania to an overactive BAS [15], [22], [31]. We also propose the existence of an additional negative feedback mechanism, stabilising the system around normal activity levels. This negative feedback loop is expressed as
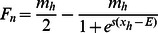
(3)where 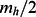 is the maximum rate of the feedback, 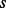 is the nonlinearity parameter for the negative feedback and 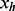 is the level of 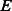 at which half of the maximum rate is reached, which in our system is defined to be the normal activity level 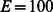, in the following referred to as 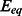. The nonlinearity parameter 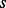 in the negative feedback loop has a similar role for the shape of this response function as 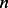 has for 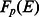, such that higher values for 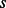 lead to sigmoidal response functions, whereas lower values approach a linear response function. We choose 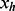 and 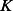 to have the same value. Moreover, the decay parameter 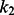 is defined to be at a value which allows the positive feedback to be at equilibrium on 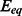 at 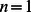 (no nonlinearity), such that



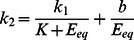
(4)If the nonlinearity parameter 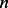 is increased to a level which allows for bistability in the auto-regulatory feedback system, the unstable fixed point lies at 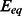. By introducing the negative feedback, this instability is locally stabilised. This leads to tristability with a high, low and medium activity level (see figure 1 and figure S2). Speculatively, this negative feedback could correspond to behavioural attempts by individuals to avoid extreme levels of behavioural activation (in other words, implementation of active coping strategies [32]). The limited effectiveness of such a mechanism is accounted for by saturating this feedback on its upper and lower bounds, such that it is not sufficient to equilibrate activation levels that are beyond a threshold value that is defined by the dynamical properties of the system (figures 1 and 2). The susceptibility of the BAS system to events of reward or frustration is accounted for by introducing the variable 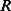, which feeds in and is consumed by levels of 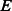 with the rate 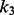. Note in this context that frustrating events do not need to be associated with negative directions in 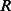, such that frustrations could also increase 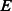. We however tested negative and positive directions in 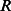 to ensure that all potential external effects on the regulation of 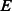 in our system could be accounted for. Therefore, in the following we refer to the effect of 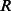 on levels of 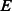 as inhibiting or activating events rather than as events of reward or frustration. The complete system is thus written as.

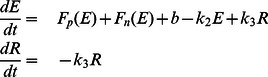
(5)All typical parameter values employed in the simulations are given in table 1.

**Table 1:** Parameter values in the model.

Parameter	Value	Unit
*k_1_*	1.5	*hr^−1^*
*k_2_*	0.0125	*hr^−1^*
*k_3_*	5	*hr^−1^*
*b*	0.5	*hr^−1^*
*K*	100	–
*m_h_*	0.26	–
*s*	5	–
*x_h_*	100	–

### Simulation

The system of differential equations was numerically solved using Scientific Python [33] and its implementation of the LSODA solver (scipy.odeint). For stochastic simulations, the Euler-Maruyama scheme [34] was applied by adding a noise term to each equation, defined by the Gaussian distribution 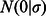 and scaled by the square root of the timestep size, 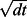. Inhibiting and activating events are chosen to occur spontaneously with the probability 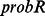, and the amplitude of these events is defined to take a value from a continuous random distribution between 0 and the maximum event amplitude 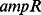. For an analytic description of the time-series data generated by simulations, we introduce the measure 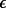, which is an indicator for the “episodicity” of the activity dynamics. By moving an averaging window with the length of 7 days over the time series data, the number of times in which the average activation level in this window is within defined bounds is recorded. Subsequently, the “episodicity” of a solution is defined as
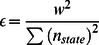
(6)where 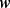 is the number of averaging windows, corresponding to the total duration of the simulation in days minus the size of the window 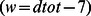, and 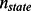 is the amount of occasions at which one of three activity levels is detected: low activity or depression is defined for window averages below 80, high activity or mania is defined for window averages above 120, and medium activity is defined for values between 80 and 120. These values are arbitrary units on our activation level scale. By doing so, we are able to distinguish between solutions that show a high variance, captured by the global signal to noise ratio SNR of the time-course data, and which still stay around one steady state of the system; and solutions that frequently switch between steady states.

For time-series data of single individuals, single stochastic simulation runs were recorded. For analysis of dynamical features, such as return to baseline 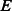 levels after activating or inhibiting external events or measures such as episodicity, the signal-to-noise ratio or the number of switching events per simulation, multiple simulations were performed for each parameter set and the average outcome for each respective analysis was recorded. If not explicitly stated otherwise, the parametric noise value 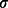 was set to 0.1 and the probability for external events to occur, 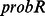 was set to 0.015. The maximum amplitide of external events occuring, 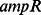, was set to vary between [−20, 20].

## Results

Our main hypothesis is that the degree of nonlinearity in the auto-activation feedback system (the BAS) is a proxy for the stage of illness, such that a value of 1 for the nonlinearity parameter 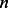 corresponds to an unaffected individual and higher values of 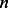 stand for an increased propensity for developing the illness. Once the individual is already bipolar, even higher values of 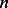 may correspond to the number of previous depressive or manic episodes. It has to be noted, however, that our model does not make predictions about how this progression in nonlinearity occurs, and therefore only captures the behaviour of the modelled system at set level of 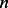. In the bifurcation analysis of our model the critical values of 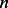 are shown to correspond to mono- and multistable solutions of the system as a function of 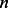 (see figure 1A). At low values of 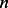, the system is monostable, whereas with increasing 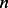 bifurcation into bistability and further into tristability happens. The influence of changes in parameter values other than 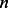 is shown in figure 1B. Parameters 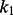, 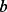 and 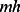 determine the level of 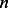 at which bifurcation occurs, and the activity maxima that the system allows for. Parameter 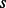, the nonlinearity parameter in the negative feedback standing for behavioural counteraction of mood swings, determines the range of stability for the branch at medium activity levels: Increasing 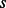 extends this range to higher 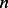, but also decreases the range between this middle stable branch to the neighbouring unstable branches at the onset of tristability, compared with lower 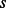 values.

We expect our model to be robust against parameter changes insofar the potential for multistability is given and the middle stable branch in the bifurcation diagram remains stable. For example, if the parameter 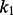 were decreased, bifurcation of the system would happen at higher levels of 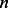, as can be seen from the outcome of our bifurcation analysis in figure 1, and thus higher levels of 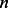 would be needed to compensate this effect for maintaining a qualitatively similar simulation output.

While auto-regulation of behavioural activity by the negative feedback loop is effective for all values of 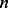, the attractor for this regulator is identical to the attractor for the positive feedback loop prior to bifurcation. This can be interpreted as a tendency of avoiding fluctuating activity levels related to mood swings that is only observable in bipolar patients already having experienced extreme changes in behavioural activity.

### Number of Previous Bipolar Episodes is Associated with a Slower Return to Baseline Mood After Disturbing Events

**Figure 3:**
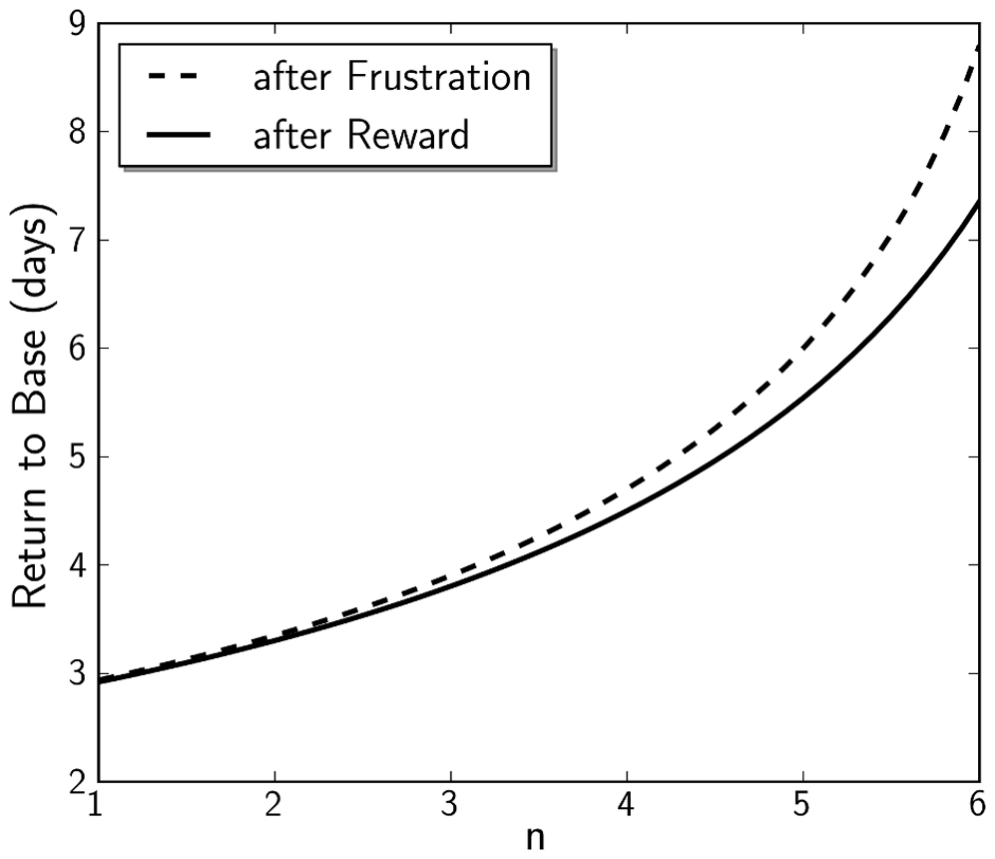
Return to base engagement levels after inhibiting and activating events in deterministic simulations with different values for the nonlinearity parameter 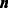. Disturbances were of magnitude 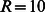 (activating events) or 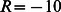 (inhibiting events) The solution is defined to have returned if it has fallen under the 1% threshold difference to the base level BES.

**Figure 4:**
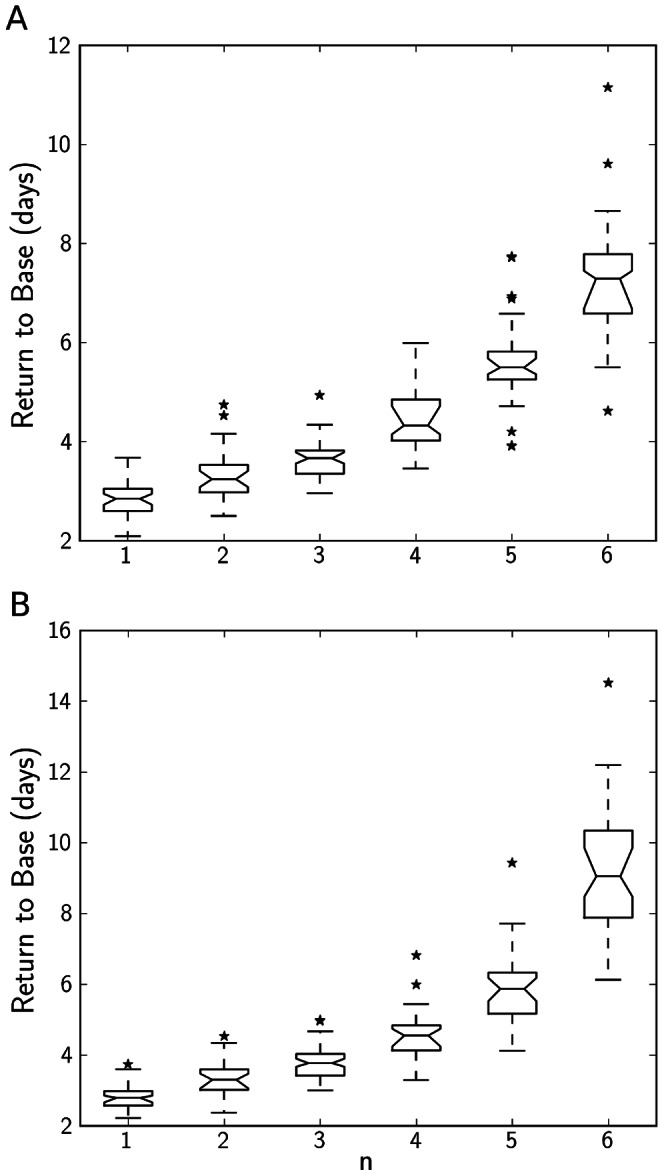
Return to base level 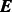 levels after activating and inhibiting events in stochastic simulations with different values for the nonlinear parameter n. A: return to 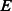 base levels after activating events, B: return to 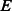 base levels after inhibiting events. Boxplots show the outcome of 50 individual runs per 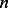. Disturbances were of magnitude 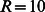 (activating events) or 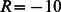 (inhibiting events) The solution is defined to have returned if it has fallen under the 1% threshold difference to the base activity levels.

Our model is able to reproduce the results that as number of previous bipolar episodes increases, so does time taken to recover from frustrating or rewarding events, solely by increasing the nonlinearity parameter 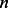 (see figures 2 and 3). This indicates that only by changing nonlinearity in the system, we are able to capture typical response behaviours for several stages in bipolarity, such as found in a previous empirical study [14]. Time series data from this study were re-analysed to infer rate constants for the decay of activation levels employed by our model, which is expressed by the parameter 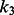, justifying exponential decay of activation levels in the time domain after disturbing events (figure 2). For this, only the top 20 percent of frustrating or rewarding events recorded in the data were taken from empirical data based on self-report questionnaires (for methods of data collection see [14]). Of these data, the BES (Behavioural Engagement Scale, expressing a scale of activation level) of the actual and following days of the event were traced until the return to the median BES, to give as the time of return to baseline, in terms of number of days. The empirical data points shown in figure 2 represent a normalised curve of the amount of individuals still above their median BES after a rewarding or frustrating event. The time trajectory of these data show a clear exponential decay, justifying our use of 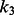 which mirrors exponential decay of perturbed 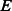 levels after external activating or inhibiting events. In the simulations, return to baseline was defined as the time taken after disturbance of activation levels by a inhibiting or activating event to fall below the threshold of mean BES 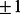
%. Disturbance in behavioural activity was 10 units on our scale of activity levels in these simulations. The time course data of our simulation show a similar behaviour in activation level decay, compared to the empirical data (figure 2). In general, for a larger range of 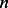, we find the trend of increasing time to return to baseline after disturbing events both for events of inhibition and activation (figure 3). This value increases exponentially towards values of 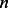 that lead the system to bifurcation, at which point disturbances can kick the solution to a higher or lower stable branch. A further set of simulations using the stochastic version of our model yielded similar results, confirming the trend of slower return to baseline for higher degrees of nonlinearity in the auto-activation feedback loop (see figure 4).

### Stochastic Simulations of Behavioural Activity Regulation Reveal Realistic Time-course Data

**Figure 5:**
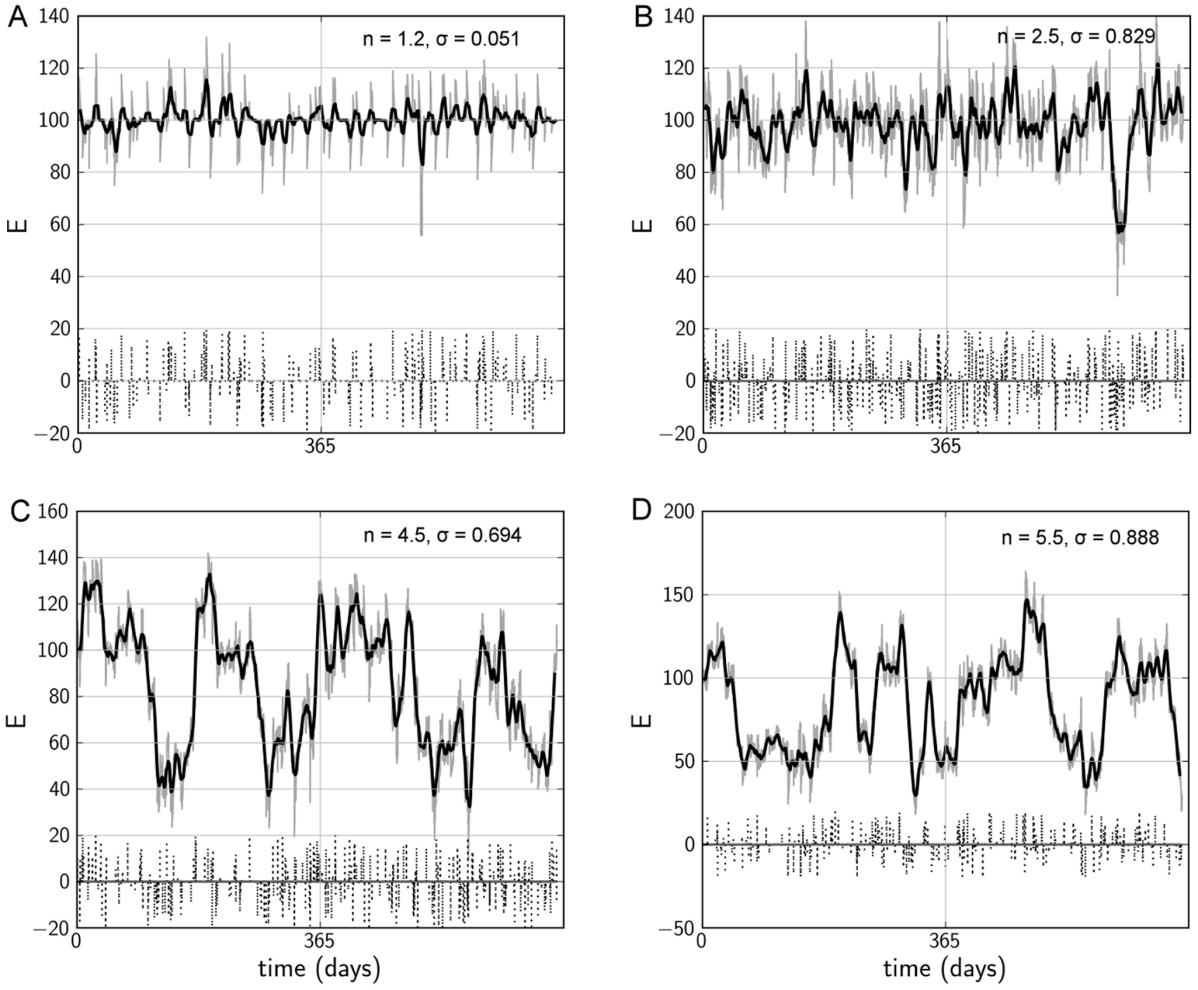
Typical outcomes of stochastic simulations for different settings of 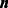 with different settings for 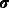. From A to D the “episodicity” value 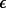 increases (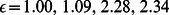), an indicator that the time course captures real episodes and not just fluctuations in behavioural activity. Gray lines are simulated activity levels, black lines are moving averages with a window of 7 days, dotted lines signify activating and inhibiting events. The time course spans 2 years.

Given different settings of parametric noise 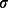, event noise (the maximum amplitude of inhibiting and activating events externally influencing the system) and nonlinearity, our stochastic simulations are able to reproduce realistic time course data of mood dynamics for normal individuals and bipolar patients (figure 5). For lower levels of 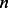, activity remains stable around a medium level, despite regular changes due to parametric and event noise. At levels for 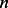 which lead the system to bifurcation, events are most likely to drive jumps in behavioural activity, whereas parametric noise is sufficient to achieve this at higher 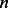. Our model is able to reproduce mood patterns that are not truly oscillatory, yet showing cyclic and regular shifting between extreme mood levels and intermediate mood.

### With Increasing Nonlinearity, Noise Moves between Scales

**Figure 6:**
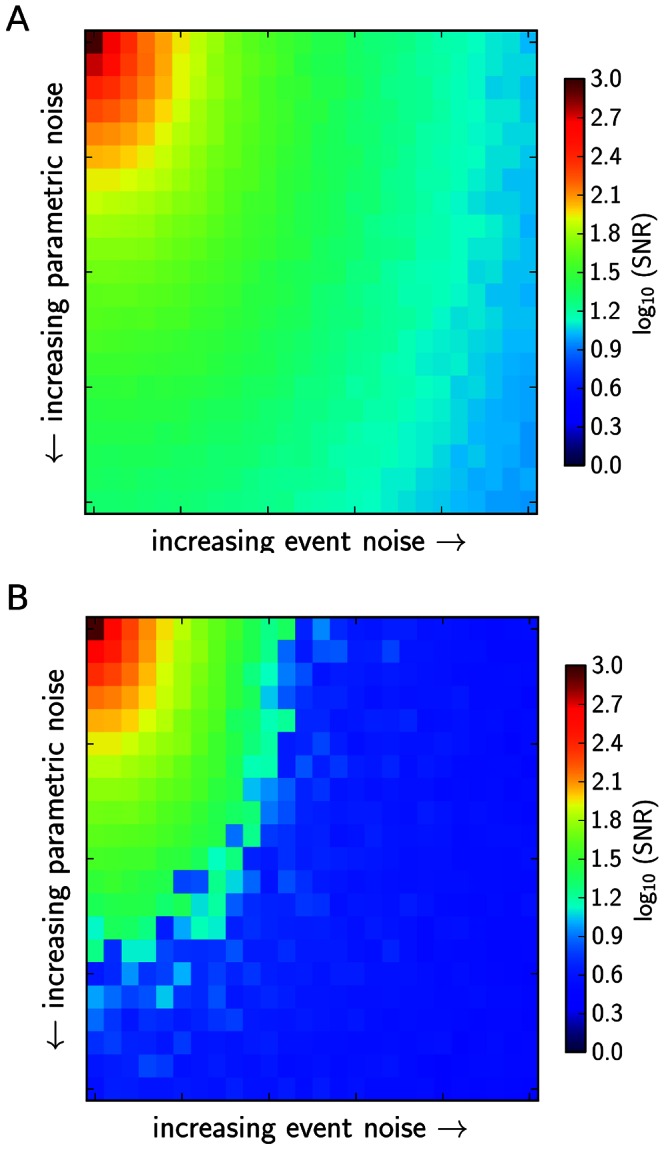
The signal to noise ratio of the solutions as a function of different settings for intrinsic and extrinsic noise in the stochastic simulations. A: The situation for 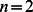, B: the situation for 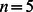. Parametric noise is the value of 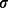 for the added Gaussian noise in the numerical integration of the system, with 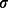 varying in the interval 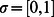. Event noise is defined as the maximum amplitude of activating and inhibiting events hitting the system, with 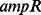 varying in [0,25]. Signal to noise ratios were averaged for 10 simulations at each parameter setting.

**Figure 7:**
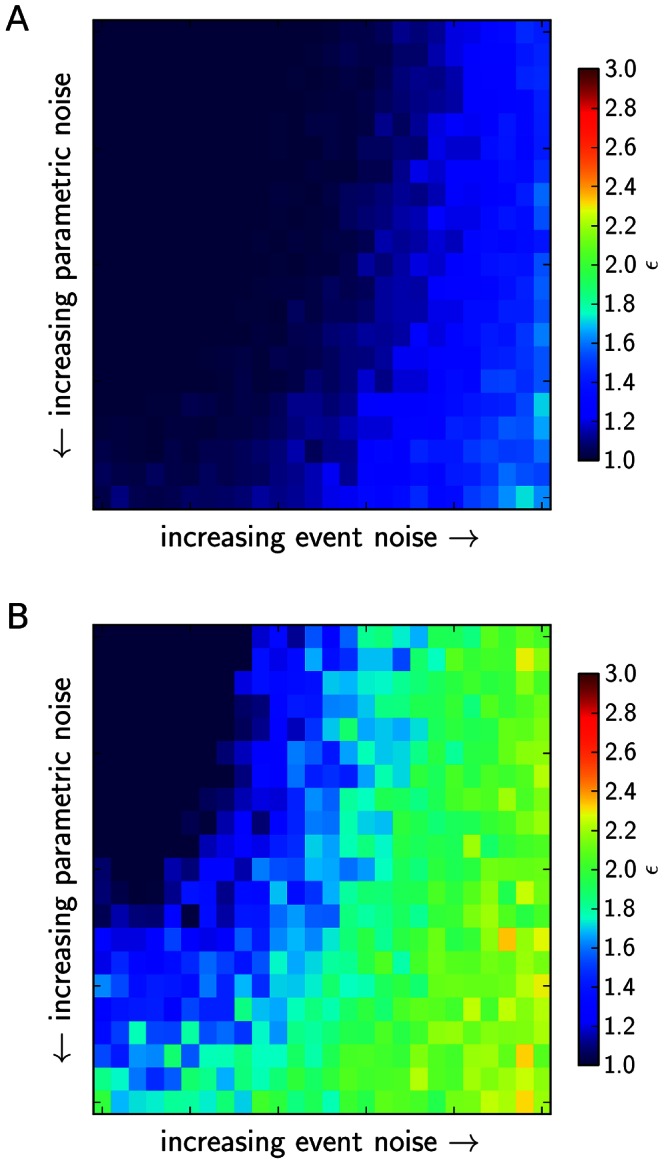
The “episodicity” 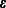 as a function of intrinsic and extrinsic noise in the stochastic simulations. A: The situation for 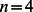, B: the situation for 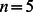. Parametric noise is the value of 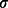 for the added Gaussian noise in the numerical integration of the system, with 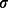 varying in the interval 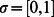. Event noise is defined as the maximum amplitude of activating and inhibiting events hitting the system, with 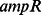 varying in [0,25]. Episodicity was averaged for 10 simulations at each parameter setting.

**Figure 8:**
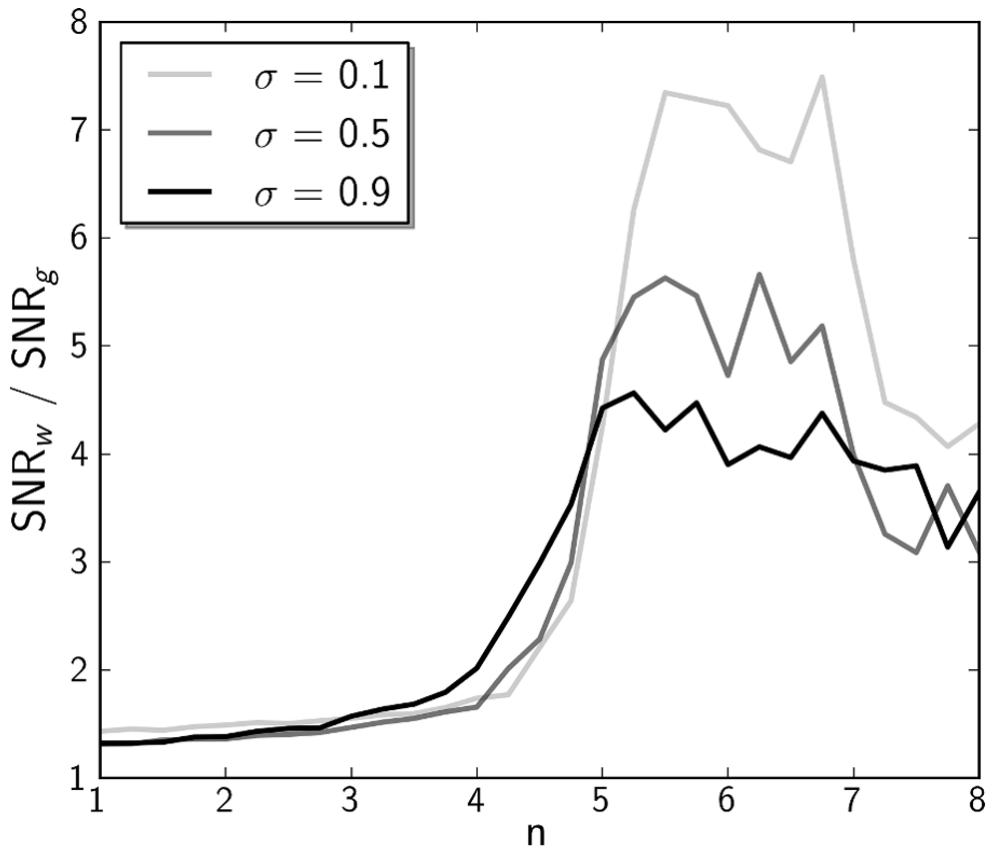
Signal-to-noise ratios on the global scale (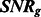, analysed on the full time scale data) versus the signal-to-noise ratios on the scale of averaging windows (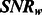, analysed on moving windows with width 7 days applied successively on the full time scale data) for given values of n with event noise 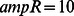. For lower 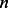, noise is similar on the global and on the smaller scales. For values of 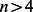, this ratio changes drastically towards lower noise on the smaller scale. This indicates that noise in the system is able to generate episodes (switch between behavioural activity states) rather than only fluctuations around one fixed point. Also, solutions are less noisy around a given steady state for 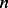 close below bifurcation values than for 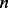 further below bifurcation values. Data points refer to the average value of SNRs, obtained by 25 stochastic simulations per value of 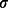, giving a duration of 2 years per time course.

Stochastic simulations under different ranges of parametric noise 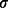, and event noise, expressed as variations of the maximum inhibiting or activating amplitude of events show that both types of noise are able to account for the increase in intrinsic system noise levels (figure 6). Also, both are able to lead to episodes, as indicated by an increase of our measure for episodicity, 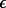. However, while intrinsic noise alone is able to generate episodes at higher 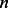, lower 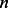 requires event noise to be high to generate switches into episodes (figure 7). Nonlinearity increases noise at a given behavioural activity level, which decreases the global signal-to-noise ratio (SNR). We calculated the ratio of this global SNR (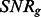) against the summed SNR on the moving average windows (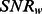). This ratio is close to zero at low 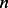 and increases rapidly as 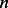 leads the system to bifurcation (see figure 8). This indicates that the level on which noise occurs switches from a smaller scale to larger scales. At below-bifurcation values for 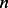, small scale noise occurs, causing various degrees of instability in activity, which seen from a dynamical viewpoint are fluctuations around one steady state. As 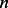 grows sufficiently large to allow for switches between several steady states, this leads to longer lasting episodes, and noise is expressed as flipping between steady state solutions, rather than as fluctuations around each of these solutions. Interestingly, this ratio drops back again for even higher values of 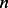. We propose that this is due to decreased global noise, since switching between states gets less frequent and episodes are longer and steadier. This is corroborated by an additional analysis that counts the switches between states during an individual stochastic simulation, following the definition of state boundaries in the episodicity measure. The distributions of switching events and episodicity for different degrees of nonlinearity are shown in figures S3 and S4.

## Discussion

Our model captures important features known from bipolar disorder such as transition between states of normal, high and low behavioural activation and traces these back to nonlinear auto-regulation feedback in underlying control systems of behavioural activity and emotional regulation. Without an exact knowledge as to what causes such behaviour mechanistically, we expect nonlinearity to be an inherent property of the control system, as is the case in most biological systems. Our model is able to reproduce typical time course evolution of normal and bipolar-type activity levels under the assumption that an increase in nonlinearity in their BAS renders an individual more prone to develop bipolar disorder. This hypothesis is further corroborated by showing that such an increase in nonlinearity is sufficient to explain slower recovery from rewarding or frustrating events individuals with a large number of previous episodes. Memory of previous states is not inherent in our model, thus progression in nonlinearity does not appear automatically during simulations. Rather, the nonlinearity parameter 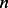 is set as a fixed parameter value for every simulation.

Our analysis of intrinsic noise with respect to parametric and event noise indicate that the positive auto-regulation of the BAS leads to an increase of noise in the system with increasing nonlinearity. Further, our analysis indicates that there are distinct scales on which the effect of noise manifests itself, and, more importantly, that nonlinearity leads to shifts between these scales. Thus, while lower nonlinearity leads to small-scale fluctuations around steady states of behavioural activation, higher nonlinearity leads to noise on the larger scale of fluctuations between extreme and intermediate activation states, together with a decreased level of fluctuations around the respective steady states. In our stochastic simulations, we distinguished between parametric noise and event noise and find that our results are not critically dependent on the source of noise insofar our measures of episodicity and SNR are concerned.

While some mathematical models of positive feedback and the effects of noise on occurrences of episodes in recurrent affective disorders have already been undertaken [7]–[9], the role of nonlinearity has not been elucidated specifically in these studies. In our model, nonlinearity is inherent in the system, expressed by auto-activation of the BAS, which is a simplification in terms of the modelling process and reduces the number of involved parameters substantially. Nonlinearity not only triggers the onset of episodes by an increase of noise in the system, but also drives the system into multistability. In contrast to the model of Huber et al., illness progression does not inevitably lead to rapid cycling and chaotic behaviour. Rather, the increase of system noise could either lead to unstable behaviour by keeping the system close to bifurcation with rapid fluctuations of behavioural activation levels, mirroring typical dynamics of cyclothymia or rapid cycling; or to a more stable behaviour and temporal fixation of activation levels in any of the three attractant steady states with a remaining susceptibility to slower variation between the three fixed points, potentially corresponding to BD-I or BD-II. This is in accordance with findings from empirical research that episode cycle lengths vary inversely with total number of cycles [35] and that there is less rapid cycling in BD-I than in BD-II [36]. Despite our model allowing for the finding that a substantial proportion of individuals with cyclothymia progress to experience BD-I or BD-II [37], we are cautious to postulate that nonlinearity increases in the time domain for every individual, thereby leading to an orderly succession along the spectrum from cyclothymia over BD-II to BD-I. In fact, our model is limited to providing a description of the system dynamics at certain levels of nonlinearity, but does not make assumptions of how the evolution of nonlinearity is structured, whether it increases or decreases continuously or in jumps, such as in the manner of a biased or unbiased random walk. Further investigations on potential candidates for the functional nonlinear relationship within the BAS will be needed to elucidate this question.

Most other mathematical models brought forward so far deal with oscillatory dynamics and therefore lack a description of how intermediate episodes or intermediate periods between bipolar episodes are possible, which is of importance for clinical research [38], [39]. Our model is able to reproduce this common phenomenon and also accounts for the finding that, while apparently cyclic in nature, episodes are not oscillatory [12], [13].

While some parameters in our model have been estimated due to lack of time-course data, some potential underpinnings of their meanings can be elucidated in the context of empirical findings. The parameter 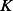, mirroring the half activation of the BAS auto-activation feedback, relates to the relative onsets of the upper versus the lower branch in our bifurcation analysis with respect to the bifurcation parameter 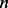. Thus, at higher levels of 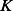, increasing nonlinearity will introduce a switch from normal to low activity levels at bifurcation, whereas at lower 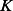 a switch to high activity levels is more likely. This seems to relate to the finding that the type of the first episode (manic/hypomanic or depressive) predicts the predominant course of following episodes [35], [36], and suggests that variations in 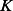 between individuals are able to capture these predominant trajectories.

Our model incorporates and predicts varying degrees of nonlinearity in BAS regulation, however its mechanistic basis remains unclear and needs further investigations. High levels of noise at degrees of nonlinearity that allow for multistability, yet also for the system to remain close to bifurcation, might correspond to a finding from BP-II individuals that inter-episode lability was higher than for unipolar depression samples [40]. Our model is also in line with findings which suggest that regulation of the BAS of bipolar patients is impaired in individuals with Bipolar Disorder [15], [22]–[25]. Recent developments in the understanding of the pathophysiology of bipolar disorder, based on neuroimaging studies, point towards potential roles of feedback pathways in prefrontal cortical neural regions implicated in emotion regulation [41]. Among other subregions within this area, the medial prefrontal cortex (MPFC) and the orbitofrontal cortex (OFC) might play a role in the reward processing activities attributed to the BAS [42]–[44]. While we currently lack sufficiently detailed knowledge about functional connectivities and dynamics of such connectivities between respective pathways to allow for conclusions about the mechanistic basis of the postulated nonlinearity in our model, we expect this nonlinearity to be dependent on the neurophysiological basis of emotional regulation and dysregulation, which are commonly associated with bipolar disorder. In a similar manner, the role of dopaminergic pathways that have been associated with the BAS [15] and the question of whether dopamine plays a role in putative increased ultrasensitivity due to high nonlinearity in the regulation of the BAS, lies outside the scope of our model and would require further research.

In conclusion, we present a mathematical model to describe a spectrum of variation in behavioural activation regulation, parts of this spectrum corresponding to the presence of clinically-diagnosable Bipolar Disorder. A strength of this model is its ability to reflect patterns revealed by observational studies of Bipolar Disorder, including the apparent non-oscillatory nature of mood swings, increasing episodicity for subtypes of Bipolar Disorder that are further along the putative spectrum, and an association between initial episode type and subsequent course of the disorder. Furthermore, the model was developed and refined with direct reference to an existing set of data concerning behavioural engagement functioning amongst individuals with and without Bipolar Disorder. A further strength of the model could also be considered a limitation: at this point the precise biological and behavioural variables corresponding to its parameters are not determined, meaning that whilst the model has considerable potential for application to multiple levels of organisation, its explanatory power is limited. Future research should seek to test predictions about the behaviour of candidate variables corresponding to the parameters of the model. This could be in terms of fluctuations in symptoms in relation to dimensions such as BAS sensitivity, implementation of coping strategies, and the action of medications, but could also be in terms of the functioning of brain areas involved in heightening approach motivation in response to signals of potential reward, and in the inhibition of such activity.

## Supporting Information

Figure S1
**A steady state analysis showing production and decay terms in the systems.** Where production (solid lines) cross degradation terms (dotted line), the system is at steady state. At higher degrees of nonlinearity (here at 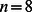), the system is tristable with three stable and two unstable steady states.(TIFF)

Figure S2
**The terms of positive feedback (dashed lines) and negative feedback (solid line) as functions of behavioural engagement levels.** Negative feedback gives a saturated function with direction towards a medium engagement (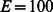).(TIFF)

Figure S3
**The distribution of episodicity for a set of 100 simulations for every value of the nonlinearity parameter 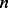.** With increasing 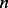, median episodicity and episodicity variance first increase, then median episodicity falls again with variance remaining high for even higher 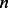.(TIFF)

Figure S4
**The distribution of switching events per simulation for sets of 100 simulations at different values of the nonlinearity parameter 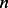.** Switching events are defined as times the average level of 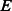 on the moving averaging window of 7 days shifts between high, medium and low states, defined as 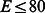 for low state, 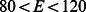 for medium state and 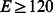 for high state. These state boundaries are also at the base of our episodicity measurement (see main text). Our analysis shows that as 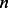 increases, the number of switching events first goes up and decreases again at values for 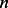 that lead deeper into the multistable regime.(TIFF)
