# Supplementary figures and images for: Relating the Bipolar Spectrum to Dysregulation of Behavioural Activation: A Perspective from Dynamical Modelling
Source: PLoS One. 2013 May 14;8(5):e63345. doi: 10.1371/journal.pone.0063345 (PMC3653950; doi:10.1371/journal.pone.0063345)

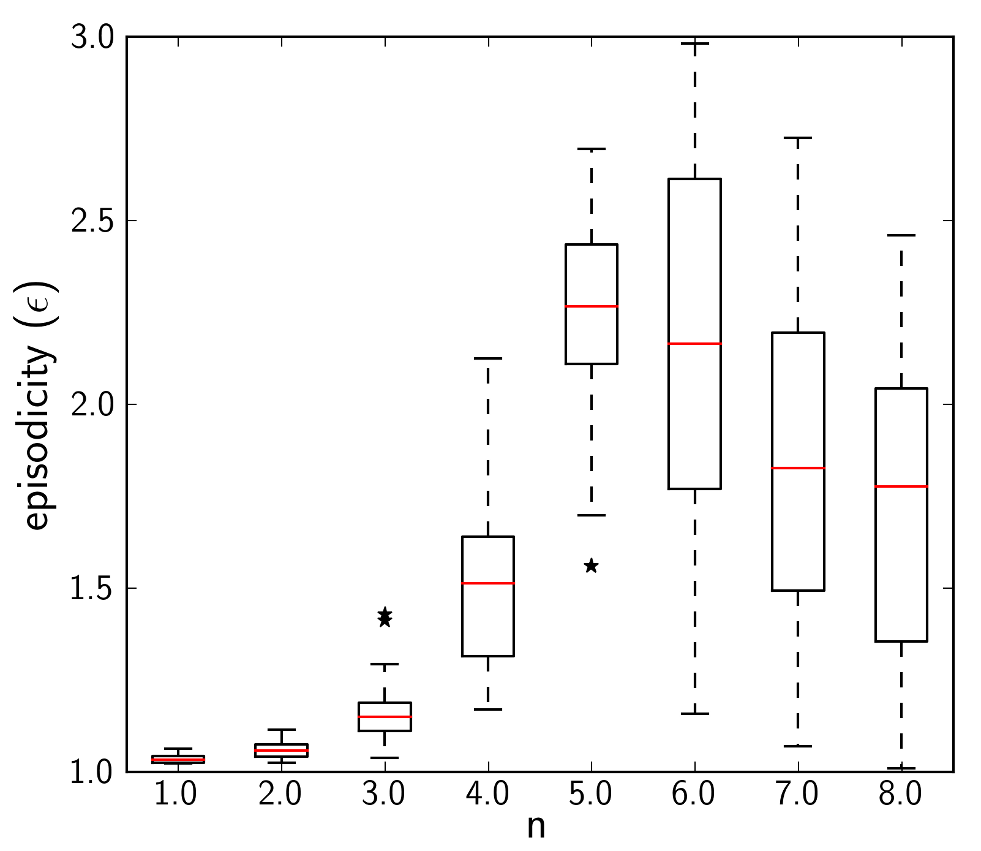

Supplement: Figure S3 — The distribution of episodicity for a set of 100 simulations for every value of the nonlinearity parameter . With increasing , median episodicity and episodicity variance first increase, then median episodicity falls again with variance remaining high for even higher . (TIFF) [file pone.0063345.s003.tif]

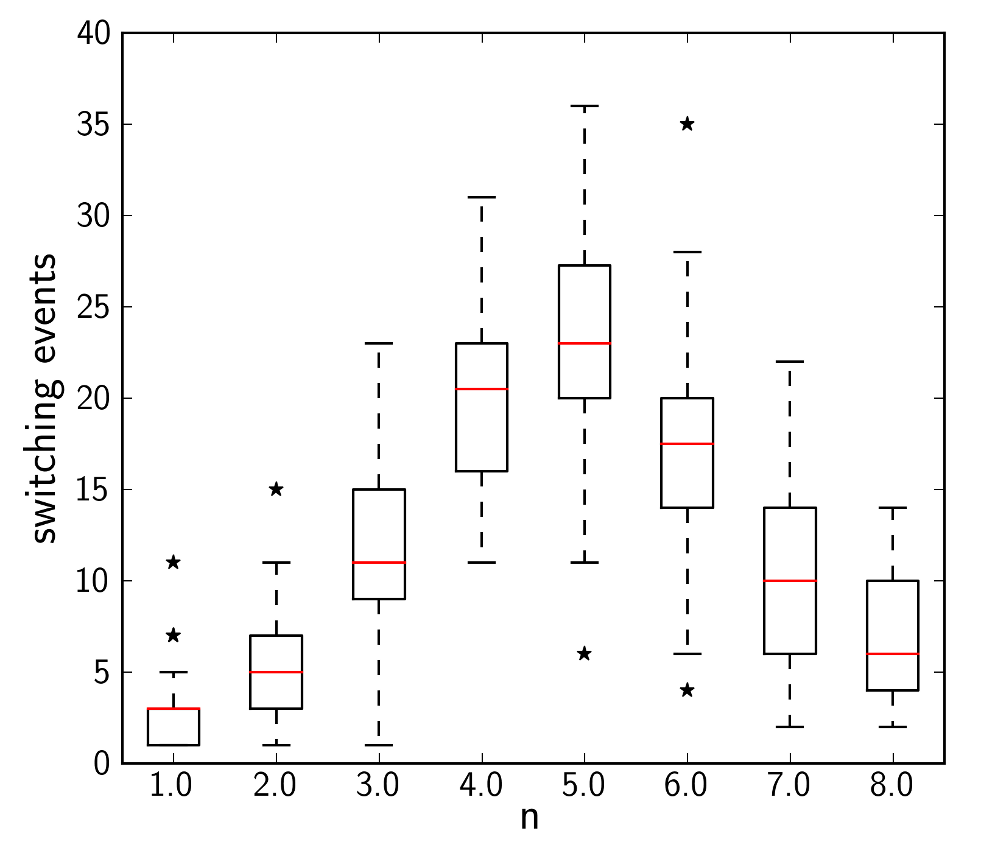

Supplement: Figure S4 — The distribution of switching events per simulation for sets of 100 simulations at different values of the nonlinearity parameter . Switching events are defined as times the average level of on the moving averaging window of 7 days shifts between high, medium and low states, defined as for low state, for medium state and for high state. These state boundaries are also at the base of our episodicity measurement (see main text). Our analysis shows that as increases, the number of switching events first goes up and decreases again at values for that lead deeper into the multistable regime. (TIFF) [file pone.0063345.s004.tif]
